# Induction of Subacute Ruminal Acidosis Affects the Ruminal Microbiome and Epithelium

**DOI:** 10.3389/fmicb.2016.00701

**Published:** 2016-05-18

**Authors:** Joshua C. McCann, Shaoyu Luan, Felipe C. Cardoso, Hooman Derakhshani, Ehsan Khafipour, Juan J. Loor

**Affiliations:** ^1^Department of Animal Sciences, University of Illinois, UrbanaIL, USA; ^2^Department of Animal Science, University of Manitoba, WinnipegMB, Canada; ^3^Department of Medical Microbiology, University of Manitoba, WinnipegMB, Canada; ^4^Division of Nutritional Sciences, University of Illinois, UrbanaIL, USA

**Keywords:** subacute ruminal acidosis, rumen, microbiome, ruminal epithelium, bacteria

## Abstract

Subacute ruminal acidosis (SARA) negatively impacts the dairy industry by decreasing dry matter intake, milk production, profitability, and increasing culling rate and death loss. Six ruminally cannulated, lactating Holstein cows were used in a replicated incomplete Latin square design to determine the effects of SARA induction on the ruminal microbiome and epithelium. Experimental periods were 10 days with days 1–3 for *ad libitum* intake of control diet, followed by 50% feed restriction on day 4, and *ad libitum* access on day 5 to the basal diet or the basal diet with an additional 10% of a 50:50 wheat/barley pellet. Based on subsequent ruminal pH, cows were grouped (SARA grouping; SG) as Non-SARA or SARA based on time <5.6 pH (0 and 3.4 h, respectively). Ruminal samples were collected on days 1 and 6 of each period prior to feeding and separated into liquid and solid fractions. Microbial DNA was extracted for bacterial analysis using 16S rRNA gene paired-end sequencing on the MiSeq Illumina platform and quantitative PCR (qPCR). Ruminal epithelium biopsies were taken on days 1 and 6 before feeding. Quantitative RT-PCR was used to determine gene expression in rumen epithelium. Bray–Curtis similarity indicated samples within the liquid fraction separated by day and coincided with an increased relative abundance of genera *Prevotella*, *Ruminococcus*, *Streptococcus*, and *Lactobacillus* on day 6 (*P* < 0.06). Although Firmicutes was the predominant phyla in the solid fraction, a SG × day interaction (*P* < 0.01) indicated a decrease on day 6 for SARA cows. In contrast, phylum Bacteroidetes increased on day 6 (*P* < 0.01) for SARA cows driven by greater genera *Prevotella* and YRC22 (*P* < 0.01). *Streptococcus bovis* and *Succinivibrio dextrinosolvens* populations tended to increase on day 6 but were not affected by SG. In ruminal epithelium, *CLDN1* and *CLDN4* expression increased on day 6 (*P* < 0.03) 24 h after SARA induction and a tendency for a SG × day interaction (*P* < 0.10) was observed for *CLDN4*. Overall, results indicate more rapid adaptation to an induced bout of SARA in the solid fraction ruminal microbiome compared with ruminal epithelium.

## Introduction

The nutrient density of dairy cattle diets has increased to maintain consistent improvements in milk yield (Plaizier et al., 2008). These dietary shifts, primarily achieved via greater concentrate inclusion relative to forage, can lead to an accumulation of volatile fatty acids in the rumen and reduced buffering capacity (Kleen et al., 2003; Stone, 2004). An overall reduction in ruminal pH such that it remains <5.6 for more than 3 h per day has been defined as subacute ruminal acidosis (SARA; Gozho et al., 2005). Compared with acute ruminal acidosis, SARA is not associated with accumulation of lactic acid in the rumen (Oetzel et al., 1999). The effects of SARA extend beyond ruminal pH and include rumen epithelial damage (Steele et al., 2011), laminitis (Cook et al., 2004), inflammation (Khafipour et al., 2009b), decreased dry matter intake (Stock and Smith, 2000; Kleen et al., 2003), lower milk yield (Stone, 1999), reduced *in situ* fiber degradation (Plaizier et al., 2001), and liver abscesses (Dirksen et al., 1985). Prevalence of SARA has been documented from 19 to 26% in early to mid-lactation cows (Garrett et al., 1997; Oetzel et al., 1999) and thus represents a significant concern for the dairy industry.

Changes in ruminal fermentation and function are the source of the multi-faceted and unfavorable consequences of SARA. Although typically described by ruminal pH, multiple reports indicate SARA effects are caused by a combination of ruminal pH and diet type (Mould and Ørskov, 1983; Russell, 1998; Calsamiglia et al., 2008; Khafipour et al., 2009a). Altering the timing and availability of dietary substrate composition may modify the bacterial community function and composition within the rumen. Understanding the shifts in the ruminal microbiome related to the observed changes in ruminal pH may uncover bacteria critical to the onset of SARA. Furthermore, effects on the microbiome may provide a more suitable definition of SARA. Advancements in high-throughput sequencing have facilitated description of bacterial communities at unprecedented detail.

Rumen epithelial tissue has many functions including nutrient absorption, metabolism, pH regulation, as well as immune and barrier functions. Impairment of barrier function has been classically linked to a decreased pH associated with periods of rapid fermentation (Gäbel et al., 1987; Aschenbach et al., 2011). The primary proteins identified in rumen epithelial tissue associated with barrier function include claudin-1 and zona occludin-1 both of which are localized in the stratum granulosum (Graham and Simmons, 2005). The molecular changes in rumen epithelium after a mild SARA bout are not well-defined. Therefore, the objectives of this experiment were to determine the effect of SARA induction on the rumen microbiome composition and predicted function in the solid and liquid fraction, describe this effect on gene expression in rumen epithelial tissue, and to link these effects with the severity of an acidotic bout.

## Materials and Methods

### Experiment Design

The experimental protocol was approved by the Institutional Animal Care and Use Committee at the University of Illinois at Urbana Champaign. Six ruminally cannulated Holstein cows were used in a replicated incomplete Latin square design. Three experimental periods consisted of 10 days with all animals receiving the same basal diet (**Supplementary Table S1**). *Ad libitum* intake was maintained for the initial 3 days of each period. On day 4, intake was reduced to 50% based on average intake from the previous 3 days. Subsequently, on day 5 all animals were given *ad libitum* access to the basal diet or the basal diet topdressed with a wheat/barley pellet at 10% of prior dry matter intake. Ruminal pH measurements were taken hourly from -2 to 22 h relative to SARA induction. Using the pH response data on day 5, cows were grouped (SARA grouping; **SG**) as Non-SARA (*n* = 7) or SARA (*n* = 5) if ruminal pH was <5.6 for more than 3 h (**Supplementary Table S2**) regardless of pellet inclusion on day 5. Data for ruminal pH, feed intake, urine pH, fecal pH, milk production have been reported previously (Luan et al., 2015). In this article, we reinterpreted the pH data in the context of effects on the ruminal microbiome and epithelium.

### Rumen Sampling and Nucleic Acid Extraction

Prior to morning feeding on days 1 and 6, ruminal contents were sampled via the ruminal cannula from the ventral sac of the rumen after mixing of the contents. Ruminal contents were squeezed through three layers of cheesecloth to separate into liquid and solid fractions. Samples were immediately put on ice and stored at -20°C prior to extraction.

DNA from the solid fraction (25 g) was extracted by homo genization followed by phenol/chloroform protocol as described by Stevenson and Weimer (2007). DNA from the liquid fraction (50 mL) was extracted using the ZR-96 Fecal DNA Kit (ZYMO Research, Irvine, CA, USA), which included a bead-beating step for mechanical lysis of bacterial cell walls. Extracted DNA from the solid fraction was standardized to 8 ng/μL for quantitative PCR (qPCR) and 20 ng/μL for Illumina sequencing. Extracted DNA was stored at -80°C for later use.

Rumen epithelium biopsies were taken on days 1 and 6 of the study prior to morning feeding. Ruminal contents were evacuated from the ventral sac allowing retraction of the epithelium approximately 6–9 inches below the ruminal cannula (Kelly et al., 1995). Papillae biopsies were excised, washed with PBS, immediately frozen in liquid nitrogen, and stored at -20°C until extraction. Rumen epithelium tissue samples were weighed and 0.4–0.6 g were subjected to RNA extraction using ice-cold QIAzol Lysis Reagent and the miRNeasy Mini Kit (Qiagen, Valencia, CA, USA) following the manufacturer’s instructions. All samples were treated with DNaseI (Qiagen, Valencia, CA, USA) to remove genomic DNA and quantification was determined using a Nanodrop ND-1000 (Nanodrop Technologies, Rockland, DE, USA). The quality of extracted RNA was evaluated using the Agilent 2100 Bioanalyzer (Agilent Technologies, Santa Clara, CA, USA) with an average RNA integrity number = 8.3 (minimum RIN = 7.4). Complementary DNA was synthesized using 100 ng RNA, 1 μg dT18, 1 μL 10 mmol/L dNTP mix (Invitrogen, Corp., Carlsbad, CA, USA), 1 μL random primers (Invitrogen, Corp., Carlsbad, CA, USA), and 10 μL DNase/RNase free water. The mixture was incubated at 65°C for 5 min and kept on ice for 3 min. A total of 6 μL of master mix composed of 5.5 μL 5X Reaction Buffer, 0.25 μL (50 U) of RevertAid^TM^ Reverse Transcriptase (Fermentas, Inc., Hanover, MD, USA), and 0.25 μL of RNase Inhibitor (10 U, Promega, Fitchburg, WI, USA) was added. The reaction was performed in an Eppendorf Mastercycler^®^ Gradient using the following temperature program: 25°C for 5 min, 42°C for 120 min, and 70°C for 15 min.

### Bacterial Quantitative PCR

Primers utilized for bacterial qPCR are listed in **Supplementary Table S3** and were validated using gel electrophoresis and Sanger sequencing. Each 10 μL reaction consisted of 4 μL sample DNA, 5 μL 1× SYBR Green with ROX (Quanta BioSciences, Gaithersburg, MD, USA), 0.4 μL each of 10 μM forward and reverse primers, and 0.2 μL DNase/RNase free water in a MicroAmp^TM^ Optical 384-Well Reaction Plate (Applied Biosystems, Foster City, CA, USA). All reactions were performed using an ABI Prism 7900 HT (Applied Biosystems, Foster City, CA, USA) using the following conditions: 5 min at 95°C, 40 cycles of 1 s at 95°C and 30 s at 60°C except an annealing temperature of 56°C used for eubacterial primer 3. The presence of a single PCR product was verified with an additional dissociation stage. All reactions were run in triplicate. Relative abundance of bacterial species was calculated using the geometric mean of two universal primers (Maeda et al., 2003; Fliegerova et al., 2014) with the efficiency-corrected Δ^-CT^ method (Ramirez-Farias et al., 2009). A portion of the 16S gene corresponding to the target of the eubacterial primer 3 (Muyzer et al., 1993) was commercially synthesized (IDT, Coralville, IA, USA). A standard curve from 9.5 × 10^7^ to 3.0 × 10^4^ molecules per μL was used to obtain the 16S copy number from each sample. Samples were diluted to 1 ng/μL for suitable qPCR performance.

### Library Construction and 16S rRNA Gene Sequencing

Amplification of the V4 region of the 16S rRNA gene used modified F515/R806 primers as described by Caporaso et al. (2012). The reverse PCR primer was indexed with 12-base Golay barcodes to facilitate multiplexing of samples. The PCR and sequencing protocol has been previously described in detail (Derakhshani et al., 2016). The 150 bp paired-end sequencing reaction was performed on a MiSeq platform (Illumina, San Diego, CA, USA) at the Gut Microbiome and Large Animal Biosecurity Laboratories, Department of Animal Science, University of Manitoba, Canada. The sequencing data were deposited into the Sequence Read Archive (SRA) of NCBI^1^ and can be accessed via accession number SRR3271885.

### 16S Read Analysis

Overlapping paired-end Illumina fastq files were merged using the PANDAseq assembler (Masella et al., 2012). All the sequences with low quality base calling scores as well as those containing uncalled bases (N) in the overlapping region were discarded. The subsequent fastq file was processed using the QIIME pipeline v1.8 (Caporaso et al., 2010b). Assembled reads were demultiplexed according to the barcode sequences, chimeric reads were filtered using UCHIME (Edgar et al., 2011), and reads were clustered into OTU (operational taxonomic units) *de novo* based on 97% similarity with UCLUST (Edgar, 2010). Representative sequences from each OTU were assigned a taxonomy using RDP Classifier (Wang et al., 2007) and aligned to the Greengenes reference database (McDonald et al., 2012) using PyNAST (Caporaso et al., 2010a).

After sample size standardization to the smallest library size (23,000 sequences/sample), OTU richness, and alpha- and beta-diversity metrics were estimated. Alpha rarefaction curves were generated with ten sampling iterations using the Chao1 index (Chao, 1984). Between sample comparisons of diversity (beta-diversity) were calculated using the Bray–Curtis metric (Beals, 1984). Bray–Curtis distance matrices were utilized in principal coordinate analysis (PCoA) to generate two-dimensional plots in PRIMER v6 software (Clarke and Gorley, 2006). Permutational multivariate analysis of variance (PERMANOVA) was implemented to test differences in beta-diversity among SG and time.

Functional metagenomic predictions were made using the bioinformatics tool PICRUSt (Langille et al., 2013). Quality-filtered, paired-end reads were used for closed-reference OTU picking in QIIME. The resulting OTU table was used in PICRUSt version 1.0.0 and functional predictions were made to the KEGG Ontology Pathways (Kanehisa and Goto, 2000). Within PICRUSt, the 16S copy number was normalized, molecular functions were predicted, and all results were summarized into KEGG pathways.

### Rumen Epithelium Quantitative Reverse Transcription-PCR

Primers utilized for rumen epithelium quantitative reverse transcription-PCR (qRT-PCR) are listed in **Supplementary Table S4**. The primer for *IGFBP5* was designed using Primer3 (Untergasser et al., 2012) and verified using gel electrophoresis and sequencing. The reaction components, real-time machine, and conditions were the same as described for bacterial qPCR. The presence of a single PCR product was verified with an additional dissociation stage. All reactions were run in triplicate. A six point relative standard curve was used to determine gene expression. Relative quantities were normalized using the geometric mean of genes *CMTM6*, *MRPL39*, and *ERC1* (Naeem et al., 2012; Minuti et al., 2015).

### Statistical Analysis

Partial least square discriminant analysis (PLS-DA) was performed on genus level assignments to identify the effect of SG and day using SIMCA P+ 13.0 (Umetrics, Umea, Sweden). In the analysis, the X variables were bacterial genera, Y variables were either SG or day comparisons, and the data were scaled using Unit Variance. Permutation was conducted to validate the models and genera with variable influence projection values below 0.5 were removed from the final model (Li et al., 2012). The R^2^ and Q^2^ estimates were used to evaluate goodness of fit and the predictive value of the model, respectively. The PLS regression coefficients were used to identify genera significantly correlated with Y variables and used to label loading scatter plots.

Relative abundance of bacteria present at >0.1% at the phyla, family, and genus taxonomic level were evaluated and logit transformed (*z* = log[*p*/(1-*p*)]) if necessary to ensure normal distribution of the residuals, where *p* represents the relative abundance of a bacterial taxa. Bacterial relative abundance and normalized epithelial gene expression data were analyzed using the MIXED procedure of SAS 9.3 (SAS Inst. Inc., Cary, NC, USA). Terms in the model included SG, day, SG × day, and period with cow nested within square as a random effect. SARA grouping means were calculated using the LSMEANS option. Additionally, bacterial relative abundance change from day 6 to day 1 was correlated with measures of pH previously reported by Luan et al. (2015) using Pearson correlations within the CORR procedure of SAS and visualized in custom heat maps. Time < 5.8 was used for correlation analysis as pH data < 5.6 in Non-SARA cows was zero-inflated. Significance was declared at *P* < 0.05 while tendencies are discussed at *P* < 0.10.

All predicted KEGG pathways by PICRUSt were subjected to a Welch’s *t*-test in STAMP 2.1.3 (Parks et al., 2014) using a Storey false discovery rate (FDR) correction (Storey and Tibshirani, 2003). After correcting for multiple tests, 63 pathways were different (*P* < 0.05) between Non-SARA and SARA cows on day 6. These pathways were then analyzed in SAS 9.4 using the MIXED procedure with the aforementioned model. All pathways with a SG × day interaction (*P* < 0.05) are shown in the results and supplement.

## Results

A total of 1,677,722 reads were generated after quality control and chimera removal resulting in an average of about 35,000 reads per sample. Sequencing depth was not affected (*P* > 0.1) by any main effect and ranged from 23,621 to 110,941. After clustering reads at 97% similarity, an average of 2,094 OTUs were obtained for each sample. At the family and genus taxonomic levels, 81.1 and 54.9% of reads were identified within the Greengenes database, respectively. Within the liquid fraction, a SG × day interaction (*P* = 0.03) was observed for the Chao1 index as community richness was higher for SARA cows on day 1 and decreased to similar levels to Non-SARA cows on day 6 (**Table 1**). The Shannon and Simpson’s indices indicated that overall alpha-diversity decreased (*P* ≤ 0.07) on day 6 in the liquid fraction. At the community level, effects of SARA induction on the microbiome were not as strongly evidenced in the solid fraction with no change in richness (Chao1) and Simpson’s index. A SG × day interaction (*P* = 0.06) was observed for the Shannon index as alpha-diversity decreased on day 6 for Non-SARA cows but increased for SARA cows.

**Table 1:** Effect of SARA induction on alpha-diversity in the liquid and solid fraction of the ruminal microbiome.^1^

	Non-SARA		SARA			*P*-value^2^		
				
	day 1	day 6	day 1	day 6	SEM	SG	Day	SG × Day
Liquid fraction								
Chao1^3^	4030	4499	5788	5019	461	0.10	0.56	0.03
Shannon^4^	9.56	9.47	9.65	9.39	0.16	0.96	0.07	0.36
Simpson’s^4^	0.996	0.995	0.996	0.995	0.0006	0.82	0.03	0.78
Solid fraction								
Chao1	2777	2555	3080	3375	359	0.29	0.86	0.24
Shannon	8.35	7.91	8.43	9.20	0.44	0.27	0.59	0.06
Simpson’s	0.979	0.969	0.983	0.998	0.014	0.38	0.76	0.21


*^1^Non-SARA = cows (*n* = 7) in which ruminal pH was not <5.6 for 3 h on day 5. SARA = cows (*n* = 5) in which ruminal pH was <5.6 for 3 h on day 5. ^2^SG = SARA grouping of cows based on ruminal pH as Non-SARA or SARA. ^3^An alpha-diversity index that estimates the number of undiscovered species within a sample as a measure of richness. ^4^Alpha-diversity measures that take into account richness and evenness of the community within a sample.*

**FIGURE 1:**
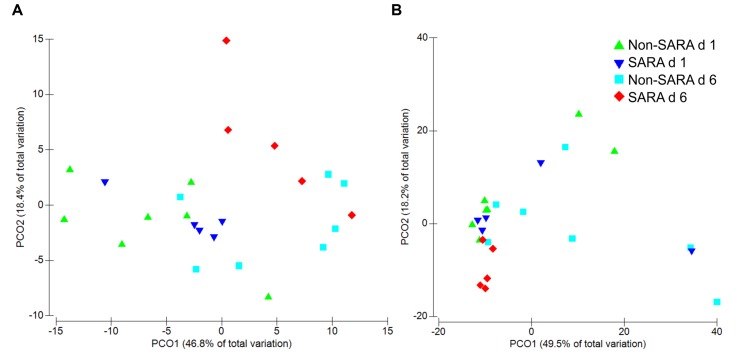
**Principal coordinate analysis (PCoA) of beta-diversity in the liquid **(A)** and solid fraction **(B)** using Bray–Curtis similarity.** Analysis by PERMANOVA revealed a day effect (*P* = 0.003), but no effect of SARA grouping (SG; *P* = 0.60) and SG × day (*P* = 0.18) was observed in the liquid fraction. In the solid fraction, PERMANOVA analysis indicated no effect of day (*P* = 0.19), SG (*P* = 0.83), or SG × day (*P* = 0.43).

Beta-diversity, measured by Bray–Curtis similarity, was visualized in principal coordinates and separated liquid fraction samples by collection day (**Figure 1A**; *P* = 0.003). Spearman correlations greater than 0.85 indicated unclassified sequences within Clostridiales and *Prevotella* were associated with the separation between days 1 and 6, respectively (data not shown). Liquid fraction samples did not cluster by SG (*P* = 0.60) and solid fraction samples did not cluster by SG or day using Bray–Curtis similarity (*P* ≥ 0.19; **Figure 1B**).

### Solid Fraction qPCR

The relative abundance of targeted bacteria species is presented in **Table 2**. A SG × day interaction (*P* < 0.04) was observed for *Anaerovibrio lipolytica*, *Prevotella bryantii*, and *Succinimonas amylolytica*. These bacteria increased on day 6 in SARA cows while no change or a decrease was observed on day 6 in Non-SARA cows regardless of day. The increase in relative abundance for *S. amylolytica* and *P. bryantii* was more than six- and four-fold, respectively. A SG effect (*P* = 0.01) was observed for *Eubacterium ruminantium* as it was greater in SARA cows. *Streptococcus bovis*, and *Succinivibrio dextrinosolvens* tended to be greater (*P* = 0.10) on day 6. The greatest value for each of these bacteria was observed on SARA day 6, but no SG effect or interaction (*P* > 0.16) was detected for *S. bovis* and *S. dextrinosolvens*. While there was no effect of SARA induction on relative abundance of *Megasphaera elsdenii* and *Selenomonas ruminantium*, *Fibrobacter succinogenes* tended to be greater (*P* = 0.08) on day 1.

**Table 2:** Effect of SARA induction on relative abundances of bacterial genera in the solid fraction using qPCR.^1^

	Non-SARA		SARA		*P*-value^2^		
			
	day 1	day 6	day 1	day 6	SG	Day	SG × Day
*A. lipolytica*	0.0024	0.0023	0.0008	0.0053	0.76	0.02	0.02
*B. proteoclasticus*	0.29	0.19	0.47	0.63	0.07	0.70	0.13
*E. ruminantium*	0.07	0.03	0.16	0.23	0.01	0.53	0.06
*F. succinogenes*^3^	0.0053	0.0010	0.0087	0.0076	0.20	0.08	0.13
*M. elsdenii*	8.1 × 10^-4^	7.2 × 10^-4^	4.3 × 10^-4^	1.0 × 10^-3^	0.94	0.41	0.27
*P. bryantii*^3^	3.1 × 10^-4^	2.1 × 10^-4^	7.6 × 10^-4^	3.4 × 10^-3^	0.09	0.22	0.04
*S. ruminantium*	0.08	0.05	0.04	0.05	0.59	0.47	0.14
*S. amylolytica*^3^	6.5 × 10^-4^	2.8 × 10^-4^	3.6 × 10^-4^	3.9 × 10^-3^	0.24	0.09	<0.01
*S. bovis*^3^	1.6 × 10^-4^	2.3 × 10^-4^	4.5 × 10^-4^	1.2 × 10^-3^	0.17	0.10	0.41
*S. dextrinosolvens*^3^	3.3 × 10^-5^	4.8 × 10^-5^	9.5 × 10^-5^	2.8 × 10^-4^	0.18	0.10	0.42
16S rRNA copy number^4^	4.6 × 10^6^	4.9 × 10^6^	4.2 × 10^6^	4.6 × 10^6^	0.75	0.39	0.97


*^1^Non-SARA = cows (*n* = 7) in which ruminal pH was not <5.6 for 3 h on day 5. SARA = cows (*n* = 5) in which ruminal pH was <5.6 for 3 h on day 5. ^2^SG = SARA grouping of cows based on ruminal pH as Non-SARA or SARA. ^3^Data were logit transformed to ensure normality of residuals. ^4^16S copy number/ng DNA.*

### Solid Fraction Microbiome Effects

Firmicutes was the most abundant phyla in the solid fraction representing 80% of all sequences while Bacteroidetes relative abundance averaged 10% (**Table 3**). Both phyla had a SG × day interaction (*P* < 0.01) as Firmicutes on day 6 decreased for SARA and Bacteroidetes increased to 23%. The effects observed within the phylum Bacteroidetes were driven by the genus *Prevotella* which averaged 77% of the sequences in the phylum. Within Firmicutes, no effects were observed for the predominant families Lachnospiraceae, Ruminococcaceae, and order Clostridiales sequences not identified at the family level (**Table 4**). Lactobacillaceae increased on Non-SARA day 6 resulting in a SG × day interaction (*P* = 0.06). Genera *Streptococcus* and *Succiniclasticum* increased on day 6 (*P* = 0.03) but were not affected by SG (**Table 5**). A SG × day interaction (*P* ≤ 0.03) with a slight decrease on Non-SARA day 6 and a larger increase in relative abundance on SARA day 6 was observed for *Clostridium*, YRC22, *Psuedobutyrivibrio*, *Anaerostipes*, and *Shuttleworthia*.

**Table 3:** Effect of SARA induction on relative abundances of bacterial phyla in the solid fraction using 16S rRNA sequencing.^1^

	Non-SARA		SARA		*P*-value^2^		
			
	day 1	day 6	day 1	day 6	SG	Day	SG × Day
Liquid fraction								
Firmicutes	31.4	28.1	27.7	29.9	0.84	0.73	0.11
Bacteroidetes	59.2	64.8	63.5	64.4	0.67	0.06	0.16
Cyanobacteria	1.35	0.55	0.65	0.25	0.31	0.02	0.43
TM-7	1.01	0.46	0.51	0.42	0.32	0.02	0.10
Actinobacteria	0.52	0.39	0.32	0.34	0.43	0.34	0.15
Proteobacteria^3^	0.18	0.10	0.12	0.13	0.95	0.22	0.16
SR-1	0.015	0.013	0.007	0.032	0.59	0.04	0.02
Solid fraction				
Firmicutes^4^	85.5	87.9	79.9	69.0	<0.01	0.01	<0.01
Bacteroidetes	4.8	3.1	9.9	23.0	<0.01	<0.01	<0.01
Actinobacteria	6.6	6.1	7.4	6.4	0.76	0.48	0.79
TM-7	2.0	1.5	2.0	0.9	0.73	0.05	0.49
SR-1	0.05	0.03	0.03	0.20	0.09	<0.01	<0.01
Proteobacteria^3^	0.02	0.01	0.05	0.04	0.18	0.64	0.95
Cyanobacteria^3^	0.05	0.03	0.02	0.01	0.23	0.14	0.93


*^1^Non-SARA = cows (*n* = 7) in which ruminal pH was not <5.6 for 3 h on day 5. SARA = cows (*n* = 5) in which ruminal pH was <5.6 for 3 h on day 5. ^2^SG = SARA grouping of cows based on ruminal pH as Non-SARA or SARA. ^3^Data were logit transformed to ensure normality of residuals. ^4^Period effect *P* < 0.05.*

**Table 4:** Effect of SARA induction on relative abundances of bacterial families in the solid fraction using 16S rRNA sequencing.^1,2^

	Non-SARA		SARA		*P*-value^3^		
			
	day 1	day 6	day 1	day 6	SG	Day	SG × Day
Bacteroidetes								
Prevotellaceae	3.29	2.42	8.13	18.32	<0.01	<0.01	<0.01
S24-7	0.63	0.33	1.12	2.01	<0.01	0.14	<0.01
Paraprevotellaceae^4^	0.18	0.06	0.32	2.35	0.02	0.34	<0.01
Bacteroidales^5^	0.39	0.22	0.37	0.73	0.45	0.44	0.03
Firmicutes								
Lactobacillaceae^4^	0.29	2.91	0.61	0.38	0.67	0.19	0.06
Streptococcaceae	0.38	1.19	0.71	0.98	0.91	0.04	0.27
Leuconostocaceae^4^	0.02	0.38	0.01	0.02	0.26	0.07	0.11
Other							
F-16	1.99	1.45	1.99	0.90	0.72	0.05	0.49


*^1^Families listed were affected by SARA induction (*P* < 0.10). Additional families unaffected by SARA induction are listed in **Supplementary Table S5**. ^2^Non-SARA = cows (*n* = 7) in which ruminal pH was not <5.6 for 3 h on day 5. SARA = cows (*n* = 5) in which ruminal pH was <5.6 for 3 h on day 5. ^3^SG = SARA grouping of cows based on ruminal pH as Non-SARA or SARA. ^4^Data were logit transformed to ensure normality of residuals. ^5^Unidentified sequences listed at the lowest level of taxonomic assignment (order).*

**Table 5:** Effect of SARA induction on relative abundances of bacterial genera in the solid fraction using 16S rRNA sequencing.^1,2^

	Non-SARA		SARA		*P*-value^3^		
			
	day 1	day 6	day 1	day 6	SG	Day	SG × Day
Bacteroidetes								
*Prevotella*	3.29	2.42	8.13	18.32	<0.01	<0.01	<0.01
YRC22^4^	0.15	0.04	0.30	2.42	0.02	0.39	<0.01
Firmicutes								
*Butyrivibrio*	16.66	12.14	12.45	12.01	0.52	0.16	0.24
*Ruminococcus*	4.92	7.37	7.27	8.09	0.54	0.11	0.41
*Lactobacillus*^4^	0.23	1.55	0.58	0.38	0.87	0.28	0.10
*Streptococcus*	0.30	1.15	0.73	1.00	0.80	0.03	0.23
*Coprococcus*	0.62	0.72	0.33	1.26	0.69	<0.01	0.01
*Moryella*	1.08	0.88	1.26	0.94	0.76	0.07	0.68
*Clostridium*	0.48	0.35	0.31	0.68	0.71	0.20	0.02
*Blautia*	0.30	0.20	0.21	0.14	0.27	0.02	0.64
*Pseudobutyrivibrio*^4^	0.07	0.02	0.07	0.42	0.18	0.57	0.01
*Anaerostipes*	0.27	0.17	0.10	0.27	0.74	0.55	0.03
*Shuttleworthia*	0.22	0.19	0.07	0.33	0.94	0.04	0.01
Other								
*Succiniclasticum*^4^	0.70	0.92	0.27	1.24	0.66	0.03	0.11


*^1^Genera listed were observed at greater than 0.1% of all solid samples. ^2^Non-SARA = cows (*n* = 7) in which ruminal pH was not <5.6 for 3 h on day 5. SARA = cows (*n* = 5) in which ruminal pH was <5.6 for 3 h on day 5. ^3^SG = SARA grouping of cows based on ruminal pH as Non-SARA or SARA. ^4^Data were logit transformed to ensure normality of residuals.*

**FIGURE 2:**
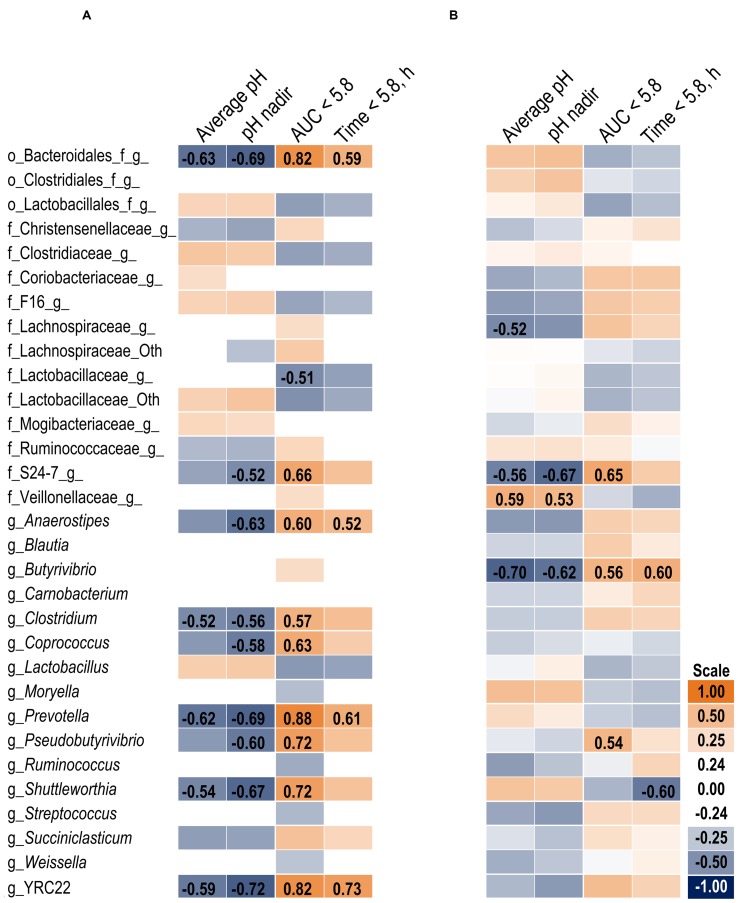
**Association heat map between the change in bacterial relative abundance over time (day 6 – day 1) and ruminal pH response on day 5 in the solid **(A)** and liquid **(B)** fractions using Pearson correlations.** All correlation coefficients greater than 0.5 or less than -0.5 are listed. The scale bar colors denote the correlation coefficients with 1 indicating a perfect positive correlation (orange) and -1 indicating a perfect negative correlation. Letter prefix denotes the lowest level of taxonomic identification [genus (g); family (f); and order (o)].

The association heat map (**Figure 2A**) supports the 16S results and also indicates the change in bacterial relative abundance from day 1 to day 6 was proportional to the severity of the acidotic bout on day 5. The strongest observed relationships are positive correlations among bacteria that increased on day 6 and greater area under the curve (AUC) below a 5.8 pH. Of the measured pH parameters, AUC < 5.8 may be the most suitable indicator of SARA effects on the microbiome within our experimental pH range.

### Liquid Fraction Microbiome Effects

At the phyla level (**Table 3**), Bacteroidetes, representing more than 60% of the sequences, tended to increase in relative abundance on day 6 (*P* = 0.06). Within Bacteroidetes, family S24-7 (**Table 6**) as well as genera *Prevotella* and YCR22 (**Table 7**) increased (*P* < 0.04) on day 6 on day 6, but unidentified sequences from order Bacteroidales decreased on day 6 (*P* < 0.01). While a SG × day interaction (*P* = 0.11) was not observed for Firmicutes, numerical trends indicated a slight decrease on day 6 for Non-SARA cows while SARA increased on day 6. This effect was realized at the family level in Lachnospiraceae (*P* = 0.01) and at the genus level in *Butyrivibrio* (*P* < 0.01). As expected, the relative abundance of *Streptococcus* and *Lactobacillus* increased on day 6 (*P* < 0.06). Collectively, in the liquid fraction many day effects (*P* < 0.05) were observed for bacterial families suggesting the impact of feed restriction and subsequent refeeding had a greater effect on microbiome composition than an acidotic bout. Correlations between the change in liquid fraction taxa and day 5 pH parameters are shown in **Figure 2B**. Relative to the solid fraction, fewer bacteria had strong correlations in the liquid fraction. Bacteria with greater correlations were also identified in the mixed model analysis with SG × day effects.

**Table 6:** Effect of SARA induction on relative abundances of bacterial families in the liquid fraction using 16S rRNA sequencing.^1,2^

	Non-SARA		SARA		*P*-value^3^		
			
	day 1	day 6	day 1	day 6	SG	Day	SG × Day
Bacteroidetes								
Prevotellaceae	40.1	49.0	43.5	46.6	0.93	0.01	0.17
S24-7	0.57	0.94	0.72	2.26	0.18	<0.01	0.04
Bacteroidales^5^	11.5	8.7	11.4	7.7	0.74	<0.01	0.56
Firmicutes								
Lachnospiraceae^4^	8.86	6.64	8.15	10.23	0.49	0.73	<0.01
Clostridiales^5^	5.62	4.27	4.75	3.09	0.33	<0.01	0.72
Christensenellaceae	1.31	0.69	0.59	0.12	0.16	<0.01	0.66
Erysipelotrichaceae	0.63	0.34	0.57	0.37	0.88	<0.01	0.20
Lactobacillales^5^	0.06	0.82	0.05	0.14	0.47	0.09	0.17
Streptococcaceae^4^	0.011	0.031	0.011	0.023	0.89	0.02	0.62
Lactobacillaceae^4^	0.008	0.075	0.018	0.026	0.94	0.09	0.20
Other								
Coriobacteriaceae	0.47	0.33	0.30	0.27	0.35	0.06	0.23
F-16	1.01	0.46	0.51	0.42	0.31	0.02	0.10


*^1^Families listed were affected by SARA induction (*P* < 0.10). Additional families unaffected by SARA induction are listed in **Supplementary Table S6**. ^2^Non-SARA = cows (*n* = 7) in which ruminal pH was not <5.6 for 3 h on day 5. SARA = cows (*n* = 5) in which ruminal pH was <5.6 for 3 h on day 5. ^3^SG = SARA grouping of cows based on ruminal pH as Non-SARA or SARA. ^4^Data were logit transformed to ensure normality of residuals. ^5^Unidentified sequences listed at the lowest level of taxonomic assignment (order).*

**Table 7:** Effect of SARA induction on relative abundances of bacterial genera in the liquid fraction using 16S rRNA sequencing.^1,2^

	Non-SARA		SARA		*P*-value^3^		
			
	Day 1	day 6	day 1	day 6	SG	Day	SG × Day
Bacteroidetes								
*Prevotella*	40.13	48.94	43.51	46.56	0.93	0.01	0.17
YRC22	1.45	1.60	1.90	2.72	0.24	0.04	0.13
Firmicutes								
*Butyrivibrio*^4^	6.27	4.23	4.99	6.62	0.71	0.58	<0.01
*Ruminococcus*	5.61	7.31	6.56	9.12	0.48	0.04	0.66
*Coprococcus*^5^	0.11	0.20	0.16	0.23	0.40	0.01	0.49
*Streptococcus*	0.011	0.017	0.014	0.025	0.66	0.06	0.55
*Lactobacillus*^5^	0.005	0.070	0.013	0.021	0.89	0.03	0.11
*Moryella*	0.008	0.008	0.014	0.009	0.65	0.56	0.61
*Anaerostipes*	0.048	0.029	0.065	0.107	0.24	0.50	0.09
*Clostridium*	0.090	0.096	0.089	0.170	0.54	0.13	0.21
*Blautia*^4^	0.049	0.037	0.047	0.044	0.88	0.29	0.46
*Pseudobutyrivibrio*	0.004	0.006	0.002	0.011	0.79	0.05	0.27
*Shuttleworthia*	0.007	0.008	0.035	0.012	0.16	0.08	0.04
Other								
*Succiniclasticum*^5^	0.143	0.189	0.222	0.342	0.40	0.09	0.69


*^1^Genera listed were observed at greater than 0.1% of all liquid samples. ^2^Non-SARA = cows (*n* = 7) in which ruminal pH was not <5.6 for 3 h on day 5. SARA = cows (*n* = 5) in which ruminal pH was <5.6 for 3 h on day 5. ^3^SG = SARA grouping of cows based on ruminal pH as Non-SARA or SARA. ^4^Period effect *P* < 0.05. ^5^Data were logit transformed to ensure normality of residuals.*

### Multivariate Analysis

**FIGURE 3:**
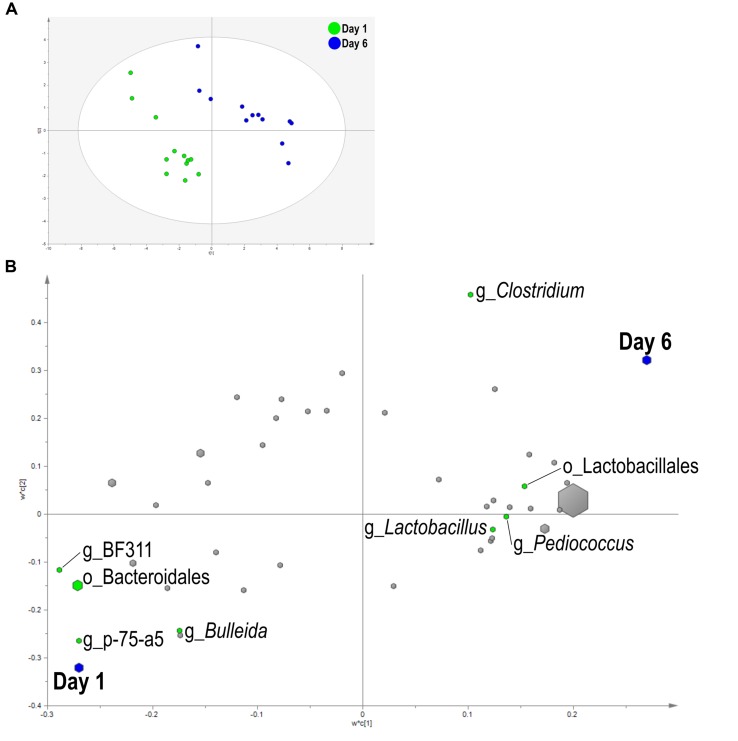
**Partial least squares discriminant analysis (PLS-DA) model of the liquid fraction bacterial communities with three components.** **(A)** PLS-DA score scatter plot discriminating between day 1 and day 6 in the liquid fraction [goodness-of-fit parameter (R^2^) (Y) = 0.97; predictive ability parameter (Q^2^) (cum) = 0.66] with each point representing a single sample. **(B)** PLS-DA loading scatter plot of bacteria classified to the lowest taxonomic level. Taxa with significant coefficient values (relationship between X and Y variables) are labeled. The size of each point corresponds to the average relative abundance of the taxa. Letter prefix denotes the lowest level of taxonomic identification [genus (g) and order (o)].

A PLS-DA was used to identify bacteria related to day and SG. Liquid fraction samples separated based on sampling day in the score plot as a three component model explained 97.1% (R^2^Y) and predicted 66.2% (Q^2^Y) of the data (**Figure 3A**). A loading score scatter plot was used to visualize specific groups of bacteria with significant coefficients in the model (**Figure 3B**). Eight bacteria had coefficients significantly different from zero that were responsible for day differences in the model; genera *Bulleida*, BF311, p-75-a5, and order Bacteroidales were enriched on day 1 while *Clostridium*, *Lactobacillus*, *Pediococcus*, and order Lactobacillales were increased on day 6. No model could be validated for an effect of SG within the liquid fraction.

**FIGURE 4:**
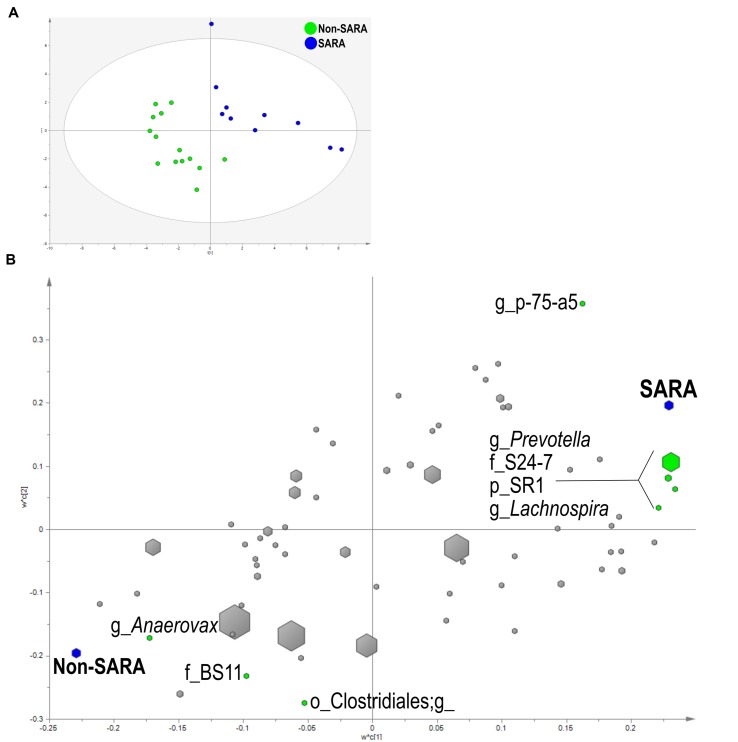
**Partial least squares discriminant analysis model of the solid fraction bacterial communities with three components.** **(A)** PLS-DA score scatter plot discriminating between Non-SARA and SARA cows in the solid fraction [goodness-of-fit parameter (R^2^) (Y) = 0.94; predictive ability parameter (Q^2^) (cum) = 0.62] with each point representing a single sample. **(B)** PLS-DA loading scatter plot of bacteria classified to the lowest taxonomic level. Taxa with significant coefficient values (relationship between X and Y variables) are labeled. The size of each point corresponds to the average relative abundance of the taxa. Letter prefix denotes the lowest level of taxonomic identification [genus (g); family (f); order (o); and phylum (p)].

Within the solid fraction, a three component the model separated the samples based on SG (**Figure 4A**). The model explained 93.9% (R^2^Y) and predicted 62% (Q^2^Y) of the data. The loading score scatter plots revealed five bacteria with significant coefficients related to SARA including *Prevotella*, p-75-a5, *Lachnospira*, family S24-7, and phylum SR1 (**Figure 4B**). Three taxa were associated with Non-SARA including genus *Anaerovorax*, family BS11, and unidentified sequences from the order Clostridiales.

### Predicted Metagenome

**FIGURE 5:**
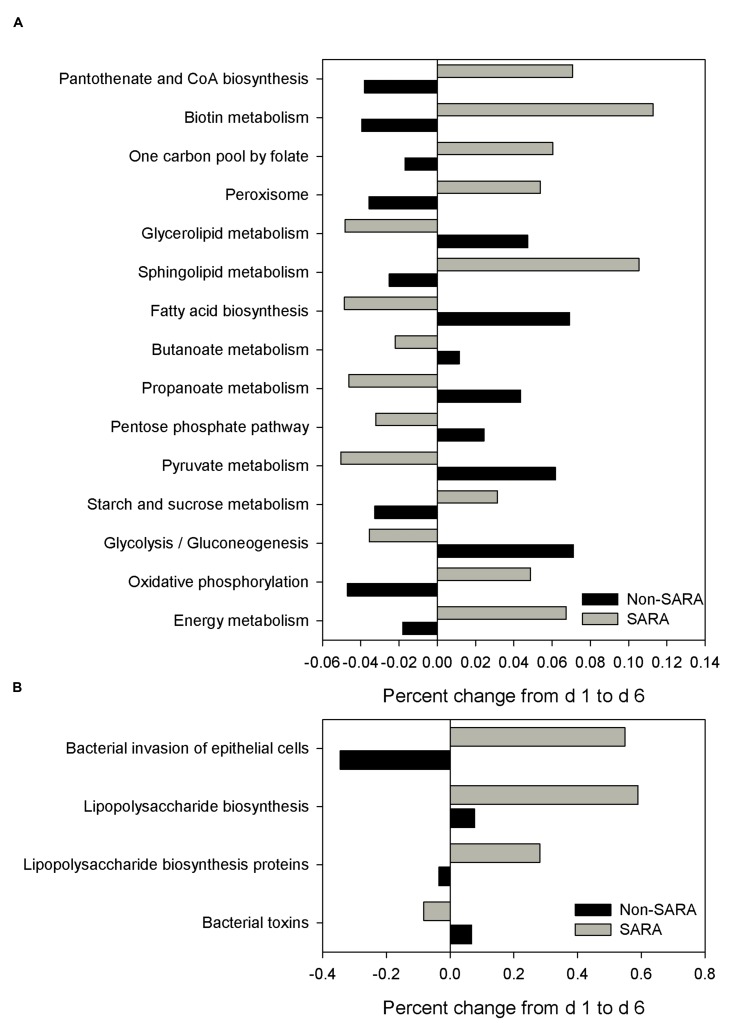
**Effect of SARA induction on the predicted metagenome pathways related to energy metabolism **(A)** and epithelial barrier function **(B)** in the solid fraction.** Values represent the percentage change in expression of a given pathway from day 1 to day 6. Positive values indicate an increased representation on day 6 compared with day 1 of a given pathway in the predicted metagenome, while negative values describe a percent decrease on day 6 of a predicted pathway.

The functional capability of the ruminal microbiome was predicted using PICRUSt to connect community composition changes in the functional profile. In the solid fraction, there were 43 affected level 3 KEGG pathways with a SG × day interaction (*P* < 0.05). The relative abundance of genes associated with the energy metabolism, oxidative phosphorylation, starch and sucrose metabolism, and sphingolipid metabolism KEGG pathways increased on day 6 for SARA compared to Non-SARA (**Figure 5A**). Pathways for bacterial invasion of epithelial cells, lipopolysaccharide (LPS) biosynthesis and proteins were also increased in SARA on d6 (**Figure 5B**). Conversely, bacterial pathways for glycolysis/gluconeogenesis, pyruvate metabolism, propanoate metabolism, and fatty acid biosynthesis were enriched on day 6 for Non-SARA cows compared to SARA. Additional significant affected pathways are listed in **Supplementary Figure S1**. Analysis of liquid fraction samples did not elucidate any differences with the predicted metagenome with no difference between SG on either day (data not shown).

### Ruminal Epithelium Gene Expression

**FIGURE 6:**
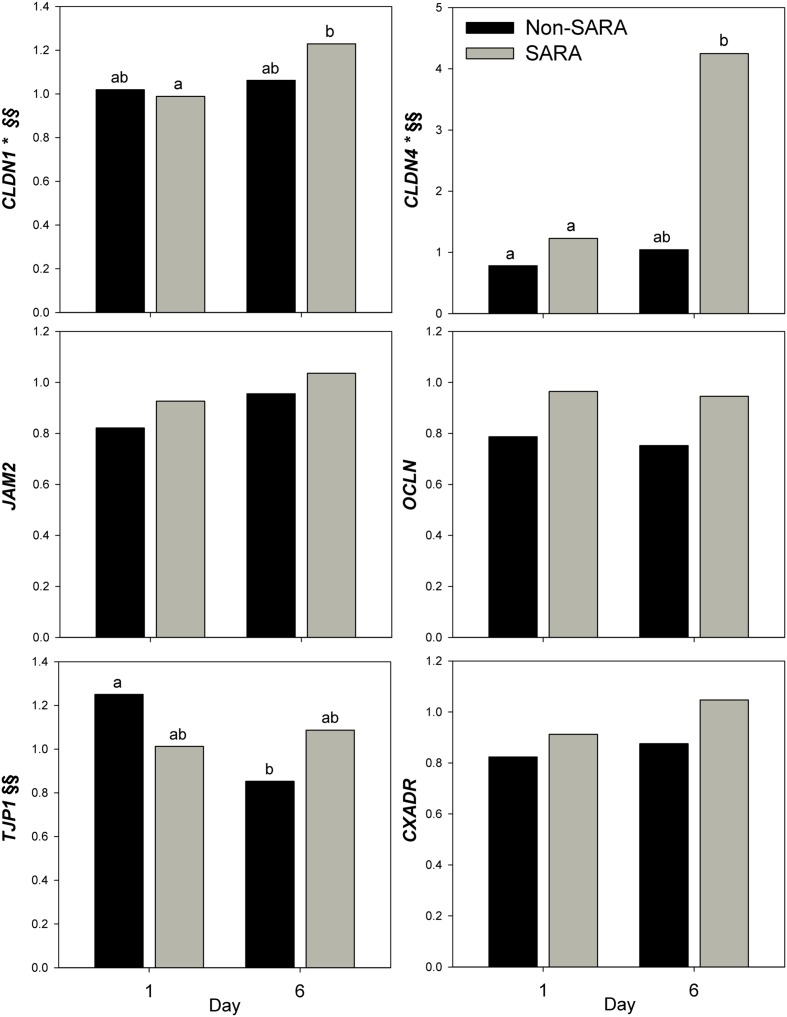
**Effect of SARA induction on barrier function gene expression in rumen epithelium tissue.** Effects (*P* ≤ 0.05) are indicated by symbols: day effect (^∗^), treatment effect (#), treatment × day effect (§), and period effect (‡). Tendencies (*P* ≤ 0.1) are indicated by symbols: day effect (^∗∗^), treatment effect (##), treatment × day effect (§§), and period effect (‡‡). Subscripts indicate pairwise differences of *P* < 0.05.

**FIGURE 7:**
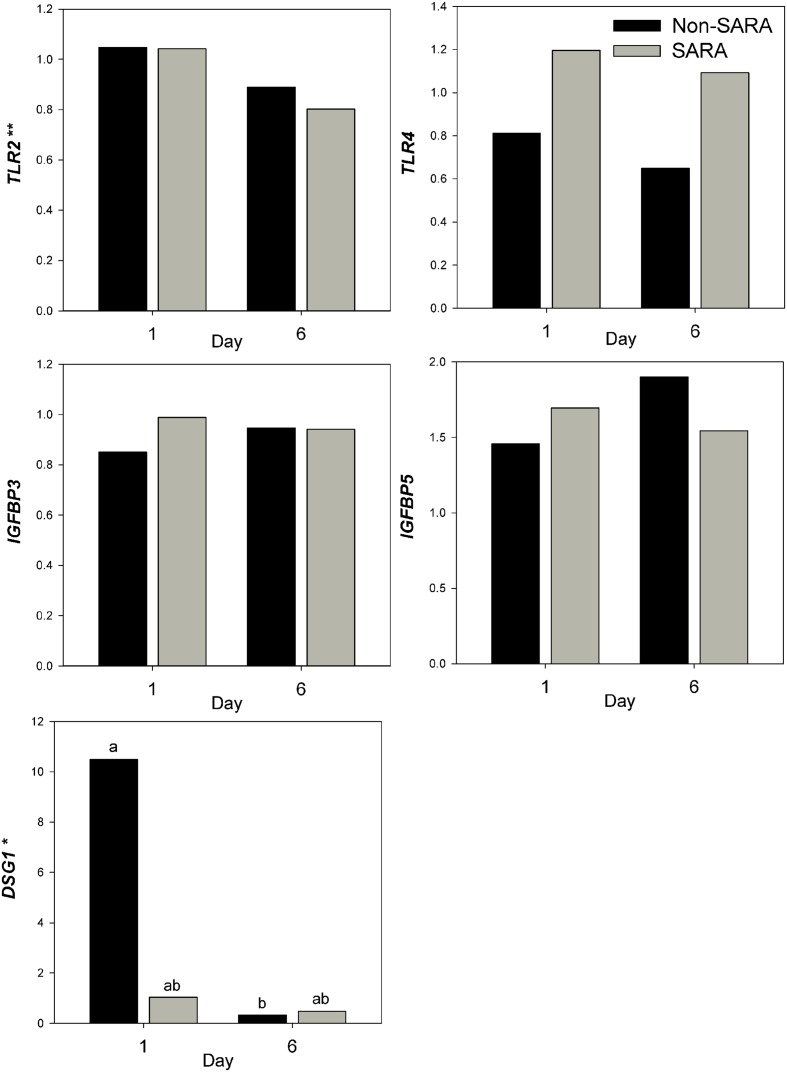
**Effect of SARA induction on gene expression in rumen epithelium tissue.** Effects (*P* ≤ 0.05) are indicated by symbols: day effect (^∗^), treatment effect (#), treatment × day effect (§), and period effect (‡). Tendencies (*P* ≤ 0.1) are indicated by symbols: day effect (^∗∗^), treatment effect (##), treatment × day effect (§§), and period effect (‡‡). Subscripts indicate pairwise differences of *P* < 0.05.

**FIGURE 8:**
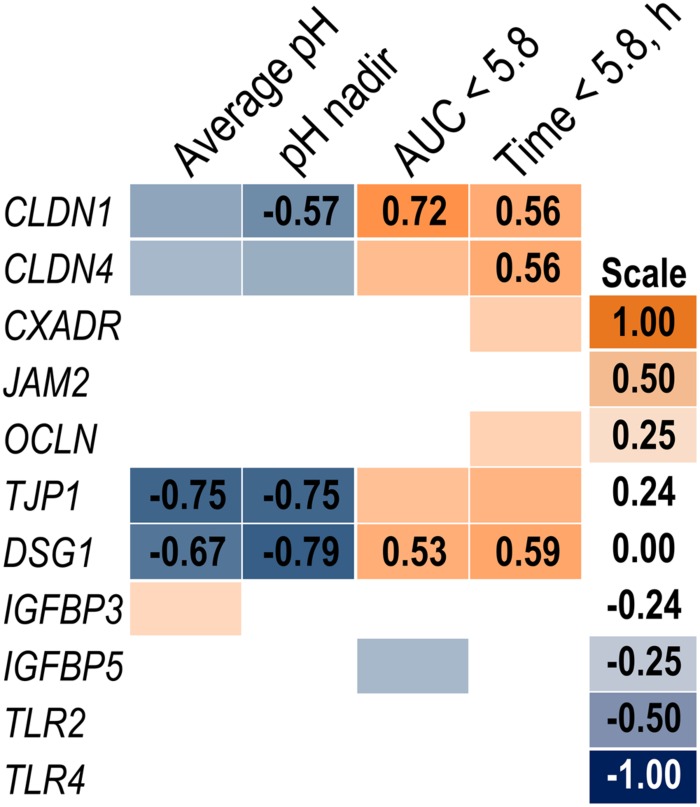
**Association heat map between the change in rumen epithelium gene expression over time (day 6 – day 1) and ruminal pH response on day 5 using Pearson correlations.** All correlation coefficients greater than 0.5 or less than -0.5 are listed. The scale bar colors denote the correlation coefficients with 1 indicating a perfect positive correlation (orange) and -1 indicating a perfect negative correlation.

Expression of genes related to barrier function in ruminal epithelium was affected by SARA induction. Claudin 4 (*CLDN4*) expression was upregulated (*P* = 0.01) on day 6 and a tendency for a SG × day interaction (*P* = 0.08) was observed with a greater increase for SARA cows on day 6 (**Figure 6**). Claudin 1 (*CLDN1*) was also upregulated (*P* = 0.03) on day 6 but the SG × day interaction (*P* = 0.10) indicated only SARA cows had greater expression on day 6. A tendency for a SG × day interaction (*P* = 0.10) was observed for Tight junction protein 1 (*TJP1*) as no change in expression was detected for SARA cows while it was down-regulated in Non-SARA cows on day 6. Relative expression of Toll-like receptor 2 (*TLR2*) decreased on day 6 (*P* = 0.05; **Figure 7**), but no day effect was detected for *TLR4* (*P* = 0.18). A day effect (*P* = 0.02) for *DSG1* indicated a decrease in expression on day 6 which was due the marked decrease for Non-SARA cows. Although, there was a tendency for Coxsackie virus and adenovirus receptor (*CXADR*) expression to be increased on day 6 (*P* = 0.10), no main effects or interactions were observed for *JAM2*, *OCLN*, *TLR4*, *IGFBP3*, and *IGFBP5*. The change in rumen epithelium gene expression from day 1 to day 6 was correlated with pH response parameters (**Figure 8**). A strong association (*R*^2^ > 0.5) was revealed between expression of *CLDN1* and *DSG1* to the pH nadir, AUC < 5.8 and time < 5.8. An increased expression of *CLDN1* and *DSG1* on day 6 (relative to day 1) positively corresponded to a proportional increase in AUC < 5.8 and time < 5.8 as well as a negative correlation to the pH nadir.

## Discussion

While current best management practices strive to minimize SARA occurrence, the continued relevance of SARA in the dairy industry is reflected in ongoing academic research. Because of the debate over the definition of SARA (Plaizier et al., 2008), the understanding of its etiology needs to be strengthened. Our objective was to elucidate effects of SARA induction using a feed restriction model on the solid and liquid fractions of the ruminal microbiome in addition to the ruminal epithelium. We defined SARA with a pH threshold of 5.6 (Gozho et al., 2005) understanding that pH is an important but not the only factor driving the onset of SARA (Calsamiglia et al., 2008). Using a *post hoc* grouping, we were able to ensure SARA and Non-SARA cows were on the same basal diet with a goal of understanding key mediators in the ruminal microbiome and epithelium 24 h after a single bout of SARA. Although, there was slight dietary variation based on the provision of a wheat/barley pellet to some cows on day 5, the inclusion of the pellet was ineffective at inducing SARA for some cows. While dietary composition and intake are primary causative agents of SARA, this study set out to understand the role of observed effects on the ruminal microbiome and epithelium during the onset of SARA.

We observed a reduction in richness for SARA cows on day 6 in the liquid fraction and a tendency for a decrease in alpha-diversity on day 6 overall. These findings correspond well with decreases in richness and diversity associated with SARA induction (Mao et al., 2013) as well as with grain feeding in general (Fernando et al., 2010). Moreover, the slight increase in richness on day 6 for Non-SARA cows suggests greater resilience in the community may be important to prevent the onset of SARA. While our results in the solid fraction were surprising as alpha-diversity increased on day 6 for SARA cows, additional evidence of greater richness post SARA induction has been observed using DGGE banding of whole rumen content samples (Lettat et al., 2012). Beta-diversity results suggest there was more variation among liquid samples compared with the solid fraction. Whereas day had a major effect characterizing the differences within the liquid fraction, more of the variation observed in the solid fraction was accounted for by the SG × day interaction. Others have reported greater variation in liquid samples when evaluating SARA microbiome changes using DGGE (Huo et al., 2014) as well as other diet types (McCann et al., 2014).

As noted by the increase in Bacteroidetes on day 6, the taxonomic evaluation of changes in the microbiome suggested the greatest effects of SARA were induced in the solid fraction. Relative abundance of Bacteroidetes was even greater on day 1 and then was only accentuated on day 6 after SARA induction. This may indicate greater levels of Bacteroidetes or *Prevotella* may predispose the rumen to the onset of SARA. Golder et al (2014) observed cows consuming higher levels of crushed wheat and ryegrass silage with greater SARA eigenvalues had greater Prevotellaceae in a predominantly liquid sample. In contrast, a longer-term induction model over 21 days with greater dietary differences resulted in lower *Prevotella* in whole rumen contents of cows with SARA (Mao et al., 2013). Evaluating the severity of acidosis during the transition period revealed a relationship between *Prevotella* in the liquid fraction and severity of acidosis (Mohammed et al., 2012), and agrees with our findings in the liquid fraction. Variation with the genus *Prevotella* has also been observed in response to SARA induction (Khafipour et al., 2009c). Understanding the undescribed diversity in the *Prevotella* genus (Bekele et al., 2010) and the limitations of taxonomic identifications with current 16S rRNA sequencing technology underscore the challenge comparing across studies given the vast differences in diet, experimental design, sampling, and methodology.

Based on pH response (**Supplementary Table S2**), the level of SARA experienced by cows in our study most closely matches the mild grain-induced SARA described by Khafipour et al. (2009c). Despite not having a group similar to our Non-SARA cows, the collection time at h 0 and control vs. induction period coincide well with days 1 and 6 in our study. Similarly to these findings, *Anaerovibrio lipolytica* and *P. bryantii* increased on day 6 relative to day 1 while *F. succinogenes* levels were not affected. The greater abundance of *F. succinogenes* observed for SARA cows was surprising given its pH sensitive metabolism (Chow and Russell, 1992) and lower abundance and activity of the cell membrane H^+^-ATPase transporter (Miwa et al., 1997). However, a similar trend for greater abundance of Fibrobacteraceae was observed in cows with greater SARA eigenvalues (Golder et al., 2014). Relative abundance of *S. bovis*, a well-described lactate producer, increased on day 6 but no effect of SG was detected despite numerical trends for a greater increase for SARA cows. Similarly, only day effects were observed at the genus level in the liquid and solid fraction. Overall increases in *S. bovis* were not related with the severity of acidotic bout within this experimental pH range which is consistent with descriptions of SARA being unassociated with lactic acid accumulation (Nagaraja and Titgemeyer, 2007). The 2.7 fold increase in *S. bovis* for SARA cows on day 6 coincided with a 2.3 fold increase in *M. elsdenii* supporting a level of synchrony between lactate producers and utilizers that may have prevented a more severe bout of SARA from developing after an abrupt feed restriction (Oetzel, 2003).

The functional capability of the rumen is more static than community composition due to functional redundancy across many community members (Weimer, 2015). In ruminants, metagenomic predictions using 16S rRNA data are comparable with shotgun sequencing data (Lopes et al., 2015). Despite a similar basal diet, energy metabolism and starch and sucrose metabolism pathways were enriched under SARA conditions which is consistent with greater glucose levels observed on higher concentrate diets with a lower ruminal pH (Ametaj et al., 2010). Significant increases in sphingolipid metabolism on day 6 in SARA cows are linked to greater relative abundance of *Prevotella*. While many gram-negative bacteria possess LPS on their cell membrane, a limited number of bacteria and fungi contain sphingolipids in their cell membrane including *Bacteroides* and *Prevotella* (Kato et al., 1995). Recent research has indicated bacterial sphingolipids are critical for survival during stressful oxidative conditions in *Bacteroides fragilis* (An et al., 2011). Although not tested under pH related stress, this mechanism may be key to the increase of *Prevotella* observed after the SARA bout. Increased LPS biosynthesis and proteins for SARA cows on day 6 corresponded well with greater levels of LPS observed with SARA induction and higher grain feeding (Khafipour et al., 2009b; Saleem et al., 2012; Mao et al., 2015) and is due to the increase of gram-negative phyla (primarily Bacteroidetes). Release of LPS from the outer membrane of gram-negative bacteria occurs during growth and stationary phases as well as during cell lysis (Wells and Russell, 1996). Pathways related to bacterial invasion of epithelial cells further suggest an increased presence of bacteria poised to take advantage of compromised barrier function in rumen epithelium. Enriched pathways related to cyanoamino acid metabolism were observed in SARA cows on day 6. This pathway is linked to beta-alanine metabolism via aspartate which connects to pantothenate and CoA biosynthesis. The coordinated enrichment of these pathways in SARA cows is supported by previous work reporting increased aspartate and beta-alanine in the rumen fluid with increased grain feeding (Saleem et al., 2012; Mao et al., 2015).

While long-term feeding of high grain diets is known to disrupt barrier function proteins (Liu et al., 2013), a single mild SARA induction did not affect epithelial barrier function determined in Ussing chambers *in vitro* (Penner et al., 2010). Claudins are tight junction proteins primarily located in the membrane of stratum granulosum cells (Graham and Simmons, 2005). Increased expression of *CLDN1* and *CLDN4* most closely coincided with a lower pH observed during SARA induction. Acidotic conditions increased expression of multiple claudins in the duodenum of rodents (Charoenphandhu et al., 2007). Although, claudins can be downregulated by the transcription factor SNAI1 (Ikenouchi et al., 2003), a mechanistic link with a low pH has not been elucidated. Desmosomes are a multi-protein complex responsible for intercellular adhesion (Holthöfer et al., 2007). Desmoglein (*DSG1*), a component of desmosomes, is highly upregulated during the recovery from an acidotic bout (Steele et al., 2011). Similarly, we observed the greatest expression levels of *DSG1* on day 1 which may represent a carry-over effect from the prior period. Toll-like receptors initiate the inflammatory response by binding to pathogen-associated molecules (Akira and Takeda, 2004). While increased expression of *TLR2* and *TLR4* has been associated with resistance to acidosis (Chen et al., 2012), this response observed in a subsequent acidosis induction following feeding a high concentrate diet for 58 days. Our results did not suggest that these adaptations occur within 24 h of a single bout of SARA. Feeding high grain diets at SARA levels over multiple weeks has been shown to increase epithelial proliferation by IGF-1 via upregulation of *IGFBP5* and downregulation of *IGFBP3* (Steele et al., 2011, 2012). The fact no effect on *IGFBP5* and *IGFBP3* was observed suggests factors unrelated to a short-term SARA induction are responsible for their regulation.

## Conclusion

These data indicate that feed restriction and subsequent SARA induction cause alterations in the ruminal microbiome and epithelium not observed in Non-SARA cows. More specifically, SARA cows had increased relative abundance of *Prevotella* and *Eubacterium ruminantium* in the solid fraction. Ruminal microbiome beta-diversity results suggest the effect of feed restriction was greater than pH differences in the liquid fraction. Predicted functional profile of the ruminal microbiome corresponded to known metabolites impacted by high concentrate feeding. Ruminal epithelium made minor adaptations 24 h after SARA including upregulation of *CLDN1* and *CLDN4*. Overall, these results extend our understanding of the rumen microbiome’s dynamic response to acidotic conditions and may facilitate targeted mediation of these events to prevent SARA.

## Author Contributions

FC designed the study. SL and FC conducted the experiment. JM and HD performed lab analysis. JM performed statistics and analyzed the data. JM, EK, and JL wrote the manuscript. All authors carefully read and approved the final version of the manuscript.

## Conflict of Interest Statement

The authors declare that the research was conducted in the absence of any commercial or financial relationships that could be construed as a potential conflict of interest.

## Footnotes

1 http://www.ncbi.nlm.nih.gov/sra
