# Supplementary material for: Induction of Subacute Ruminal Acidosis Affects the Ruminal Microbiome and Epithelium
Source: Front Microbiol. 2016 May 18;7:701. doi: 10.3389/fmicb.2016.00701 (PMC4870271; doi:10.3389/fmicb.2016.00701)
Supplement: Supplementary file 1 [file Data_Sheet_1.PDF]

Supplementary Material

Induction of Subacute Ruminal Acidosis Affects the Ruminal Microbiome and Epithelium

Joshua C. McCann*, Shaoyu Luan, Felipe C. Cardoso, Hooman Derakhshani, Ehsan Khafipour, Juan J. Loor*

***Correspondence:**

Juan Loor, Department of Animal Sciences, University of Illinois, 1207 W Gregory Dr., Urbana, IL 61801, USA; jloor@illinois.edu

Joshua McCann, Department of Animal Sciences, University of Illinois, 1207 W Gregory Dr., Urbana, IL 61801, USA; jcmccan2@illinois.edu

Table S1. Ingredient composition of the basal lactation diet.

Ingredient, % DM	
Alfalfa hay	3.36
Grass hay	2.80
Corn silage	33.6
Alfalfa silage	9.32
Cottonseed	8.01
Soy hulls	4.66
Dry ground corn grain	21.0
Lactating supplement[12]	17.3

[1] Lactating supplement was formulated for 43.6% CP, 13.4% NDF, 7.1% ADF, 0.3% lignin, 5.47% crude fat, and 25.21 mEq/100g DCAD, and contained: 24.7% soybean meal, 26.16% bypass protein, 1.94% bypass fat, 7.53% blood meal, 4.3% sodium bicarbonate, 6.13% limestone, 2.26% dicalcium phosphate, 1.18% white salt, and <1% of each of the following: trace minerals, vitamin E.

[2] Additional wheat-barley pellet was topdressed on d 4 feeding.

Table S2. Ruminal pH response parameters after SARA induction on d 5.[1,2]

	Non-SARA		SARA	
	Mean	SD	Mean	SD
No. of observations	7		5	
Average pH	6.50	0.18	6.13	0.06
pH nadir	5.90	0.21	5.38	0.09
AUC < 5.8	0.01	0.02	1.35	0.52
Time < 6.0, h	4.0	3.87	10.6	2.30
Time < 5.8, h	0.4	0.53	7.0	1.73
Time < 5.6, h	0.0	0.00	3.4	1.52

[1]Reinterpreted data from Luan et al (Luan et al., 2015).

[2]Non-SARA = cows (n = 7) in which ruminal pH was not < 5.6 for 3 h on d 5. SARA = cows (n = 5) in which ruminal pH was < 5.6 for 3 h on d 5.

Table S3. Primers utilized for qPCR of ruminal bacteria.

Bacteria species		Primers (5` - 3`)	Source
Anaerovibrio lipolytica	F	GAAATGGATTCTAGTGGCAAACG	(Minuti et al., 2015)
	R	ACATCGGTCATGCGACCAA	
Butyrivibrio proteoclasticus	F	GGGCTTGCTTTGGAAACTGTT	(Minuti et al., 2015)
	R	CCCACCGATGTTCCTCCTAA	
Eubacterium ruminantium	F	CTCCCGAGACTGAGGAAGCTTG	(Stevenson and Weimer, 2007)
	R	GTCCATCTCACACCACCGGA	
Fibrobacter succinogenes	F	GCGGGTAGCAAACAGGATTAGA	(Stevenson and Weimer, 2007)
	R	CCCCCGGACACCCAGTAT	
Megaspheara elsdenii	F	AGATGGGGACAACAGCTGGA	(Stevenson and Weimer, 2007)
	R	CGAAAGCTCCGAAGAGCCT	
Prevotella bryantii	F	AGCGCAGGCCGTTTGG	(Stevenson and Weimer, 2007)
	R	GCTTCCTGTGCACTCAAGTCTGAC	
Selenomonas ruminantium	F	CAATAAGCATTCCGCCTGGG	(Stevenson and Weimer, 2007)
	R	TTCACTCAATGTCAAGCCCTGG	
Succinimonas amylolytica	F	CGTTGGGCGGTCATTTGAAAC	(Khafipour et al., 2009)
	R	CCTGAGCGTCAGTTACTATCCAGA	
Streptococcus bovis	F	TTCCTAGAGATAGGAAGTTTCTTCGG	(Stevenson and Weimer, 2007)
	R	ATGATGGCAACTAACAATAGGGGT	
Succinivibrio dextrinosolvens	F	TAGGAGCTTGTGCGATAGTATGG	(Khafipour et al., 2009)
	R	CTCACTATGTCAAGGTCAGGTAAGG	
Eubacterial primer 1	F	GGATTAGATACCCTGGTAGT	(Fliegerova et al., 2014)
	R	CACGACACGAGCTGACG	
Eubacterial primer 2	F	GTGSTGCAYGGYTGTCGTCA	(Maeda et al., 2003)
	R	ACGTCRTCCMCACCTTCCTC	
Eubacterial primer 3	F	CCTACGGGAGGCAGCAG	(Muyzer et al., 1993)
	R	ATTACCGCGGCTGCTGG	

Table S4 Primers utilized for qRT-PCR of rumen epithelium tissue.

Gene	Accession #	Primers[1]	Primers (5'-3')	bp[2]	Source
CXADR	NM_174298.4	F.644	TCCGACTCACAGAAACTGCC	106	(Walker et al., 2014)
CXADR		R.749	CCGTACAGGTGTATGTCCCG		
CLDN1	NM_001001854	F.480	GGCATCCTGCTGGGACTAATAG	100	(Minuti et al., 2015)
CLDN1		R.579	CAGCCATCCGCATCTTCTGT		
CLDN4	NM_001014391	F.695	CCCCAGCCAGCAACTACGT	103	(Minuti et al., 2015)
CLDN4		R.797	TCACAGATTGCAGTGAGCTCAGT		
JAM2	NM_001083736.1	F.592	CCCCATCGGAACAAGGTCAA	129	(Walker et al., 2014)
JAM2		R.720	GACATCGCAGCTCTACCACA		
OCLN	NM_001082433.2	F.466	GCCATTTTCGCCTGTGTTG	101	(Minuti et al., 2015)
OCLN		R.566	CCAAAGGCACTTCCTGCATAA		
TJP1	XM_582218.8	F.2965	GCACATAGGATCCCTGAACCA	107	(Minuti et al., 2015)
TJP1		R.3071	TGCTTCCGGTAGTACTCCTCATC		
TLR2	NM_174197.2	F.2238	CTGGCAAGTGGATTATCGACAA	102	(Jacometo et al., 2015)
TLR2		R.2340	TACTTGCACCACTCGCTCTTCA		
TLR4	NM_174198.6	F.555	TGCGTACAGGTTGTTCCTAACATT	109	(Jacometo et al., 2015)
TLR4		R.664	TAGTTAAAGCTCAGGTCCAGCATCT		
IGFBP3	NM_174556.1	F.542	GCGCCCTTACCTGCTACC	86	(Grala et al., 2014)
IGFBP3		R.627	CAGCCTGGTTCTCTGTGCT		
IGFBP5	NM_001105327.2	F.513	GTCCAAGTTCGTGGGAGGAG	89	this study
IGFBP5		R.601	AGGGCCCCTGCTCAGATTTC		
DSG1	NM_174045.1	F.775	AGACAGAGAGCAATATGGCCAGT	88	(Steele et al., 2012)
DSG1		R.862	TTCACACTCTGCTGACATACCATCT		
CMTM6	NM_001035066.1	F.419	TTCACTTTGACACATGACAATACCA	103	(Minuti et al., 2015)
CMTM6		R.521	CACGGAGCATAAAGGAGAACTCA		
ERC1	NM_001205419.1	F.2981	CCTCCCATTCCGGTCAAAG	105	(Naeem et al., 2012)
ERC1		R.3085	GTCTGATGTACAACTTGAGCTTGCTT		
MRPL39	NM_001080730.2	F.602	AGGTTCTCTTTTGTTGGCATCC	101	(Bionaz and Loor, 2007)
MRPL39		R.502	TTGGTCAGAGCCCCAGAAGT		

[1]Primer direction (F = forward; R = reverse) and hybridization position on the sequence.

[2]Amplicon size in base pair (bp).

Table S5. Effect of SARA induction on relative abundances of bacterial families in the solid fraction using 16S rRNA sequencing.[1]

	Non-SARA		SARA		*P*-value[2]		
	d 1	d 6	d 1	d 6	SG	Day	SG × Day
Firmicutes							
Lachnospiraceae	33.27	24.34	27.75	27.96	0.87	0.14	0.12
Ruminococcaceae	15.05	15.40	17.29	19.54	0.44	0.54	0.65
Clostridiales[4]	16.54	14.44	15.46	13.05	0.76	0.14	0.92
Lactobacillales[4]	1.57	7.83	4.57	0.02	0.71	0.79	0.11
Mogibacteriaceae	3.94	3.64	3.41	2.61	0.42	0.26	0.61
Veillonallaceae[3]	1.84	1.67	0.59	1.41	0.38	0.24	0.14
Christensenellaceae	1.18	0.79	0.93	0.89	0.78	0.11	0.21
Clostridiaceae	1.90	1.82	1.82	1.69	0.86	0.75	0.98
Erysipelotrichaceae	0.21	0.22	0.23	0.17	0.78	0.18	0.11
Actinobacteria							
Coriobacteriaceae	6.37	5.85	7.14	6.32	0.74	0.49	0.88

[1]Non-SARA = cows (n = 7) in which ruminal pH was not < 5.6 for 3 h on d 5. SARA = cows (n = 5) in which ruminal pH was < 5.6 for 3 h on d 5.

[2]SG = SARA grouping of cows based on ruminal pH as Non-SARA or SARA.

[3]Data were logit transformed to ensure normality of residuals.

[4]Listed at the lowest level of taxonomic assignment (order).

Table S6. Effect of SARA induction on relative abundances of bacterial families in the liquid fraction using 16S rRNA sequencing.[1]

	Non-SARA		SARA		*P*-value[2]		
	d 1	d 6	d 1	d 6	SG	Day	SG × Day
Bacteroidetes							
Paraprevotellaceae	4.92	4.96	5.45	6.41	0.39	0.39	0.43
Firmicutes							
Ruminococcaceae	13.67	12.97	12.87	14.38	0.67	0.21	0.39
Clostridiaceae[3]	0.25	0.21	0.20	0.24	0.91	0.93	0.46
Mogibacteriaceae	0.48	0.39	0.41	0.41	0.78	0.44	0.41
Carnobacteriaceae	0.20	0.10	0.20	0.27	0.73	0.86	0.45
Veillonallaceae	0.16	0.22	0.24	0.34	0.49	0.11	0.86

[1]Non-SARA = cows (n = 7) in which ruminal pH was not < 5.6 for 3 h on d 5. SARA = cows (n = 5) in which ruminal pH was < 5.6 for 3 h on d 5.

[2]SG = SARA grouping of cows based on ruminal pH as Non-SARA or SARA.

[3]Data were logit transformed to ensure normality of residuals.

Figure S1. Effect of SARA induction on the predicted metagenome pathways in the solid fraction. Values represent the percentage change in expression of a given pathway from d 1 to d 6. Positive values indicate an increased representation on d 6 compared with d 1 of a given pathway in the predicted metagenome, while negative values describe a percent decrease on d 6 of a predicted pathway.

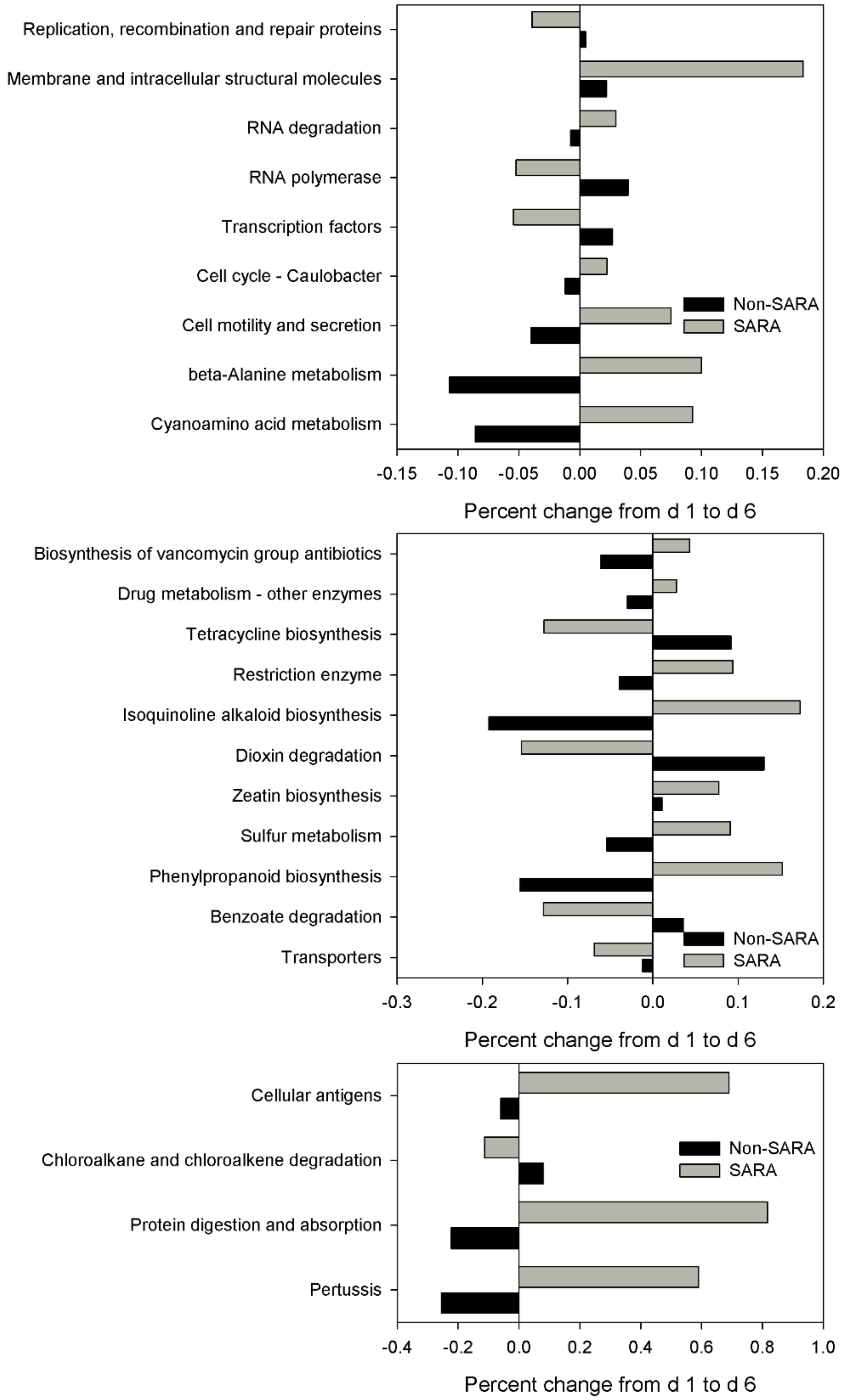

References

Bionaz, M., and Loor, J.J. (2007). Identification of reference genes for quantitative real-time PCR in the bovine mammary gland during the lactation cycle. *Physiological Genomics* 29**,** 312-319. doi: 10.1152/physiolgenomics.00223.2006.

Fliegerova, K., Tapio, I., Bonin, A., Mrazek, J., Callegari, M.L., Bani, P., Bayat, A., Vilkki, J., Kopečný, J., and Shingfield, K.J. (2014). Effect of DNA extraction and sample preservation method on rumen bacterial population. *Anaerobe* 29**,** 80-84. doi: 10.1016/j.anaerobe.2013.09.015.

Grala, T.M., Phyn, C.V.C., Kay, J.K., Rius, A.G., Lucy, M.C., Littlejohn, M.D., Snell, R.G., and Roche, J.R. (2014). Gene expression in liver and adipose tissue is altered during and after temporary changes to postpartum milking frequency. *Journal of Dairy Science* 97**,** 2701-2717. doi: 10.3168/jds.2013-7024.

Jacometo, C.B., Osorio, J.S., Socha, M., Correa, M.N., Piccioli-Capelli, F., Trevisi, E., and Loor, J.J. (2015). Maternal consumption of organic trace minerals (4-Plex®) alters calf systemic and neutrophil mRNA and microRNA indicators of inflammation and oxidative stress. *Journal of Dairy Science* 98**,** 7717-7729.

Khafipour, E., Li, S., Plaizier, J.C., and Krause, D.O. (2009). Rumen microbiome composition determined using two nutritional models of subacute ruminal acidosis. *Applied and Environmental Microbiology* 75**,** 7115-7124. doi: 10.1128/aem.00739-09.

Luan, S., Cowles, K., Murphy, M.R., and Cardoso, F.C. (2015). Effect of a grain challenge on ruminal, urine, and fecal pH, total-tract starch digestibility, and milk composition of Holstein and Jersey cows. *J. Dairy Sci (in press)*.

Maeda, H., Fujimoto, C., Haruki, Y., Maeda, T., Kokeguchi, S., Petelin, M., Arai, H., Tanimoto, I., Nishimura, F., and Takashiba, S. (2003). Quantitative real-time PCR using TaqMan and SYBR Green for Actinobacillus actinomycetemcomitans, Porphyromonas gingivalis, Prevotella intermedia, tetQ gene and total bacteria. *FEMS Immunology and Medical Microbiology* 39**,** 81-86. doi: 10.1016/s0928-8244(03)00224-4.

Minuti, A., Palladino, A., Khan, M.J., Alqarni, S., Agrawal, A., Piccioli-Capelli, F., Hidalgo, F., Cardoso, F.C., Trevisi, E., and Loor, J.J. (2015). Abundance of ruminal bacteria, epithelial gene expression, and systemic biomarkers of metabolism and inflammation are altered during the peripartal period in dairy cows. *Journal of Dairy Science* 98**,** 8940-8951. doi: 10.3168/jds.2015-9722.

Muyzer, G., de Waal, E.C., and Uitterlinden, A.G. (1993). Profiling of complex microbial populations by denaturing gradient gel electrophoresis analysis of polymerase chain reaction-amplified genes coding for 16S rRNA. *Applied and Environmental Microbiology* 59**,** 695-700.

Naeem, A., Drackley, J.K., Stamey, J., and Loor, J.J. (2012). Role of metabolic and cellular proliferation genes in ruminal development in response to enhanced plane of nutrition in neonatal Holstein calves. *Journal of Dairy Science* 95**,** 1807-1820. doi: 10.3168/jds.2011-4709.

Steele, M.A., Dionissopoulos, L., AlZahal, O., Doelman, J., and McBride, B.W. (2012). Rumen epithelial adaptation to ruminal acidosis in lactating cattle involves the coordinated expression of insulin-like growth factor-binding proteins and a cholesterolgenic enzyme. *Journal of Dairy Science* 95**,** 318-327. doi: 10.3168/jds.2011-4465.

Stevenson, D.M., and Weimer, P.J. (2007). Dominance of *Prevotella* and low abundance of classical ruminal bacterial species in the bovine rumen revealed by relative quantification

real-time PCR. *Applied Microbiology and Biotechnology* 75**,** 165-174. doi: 10.1007/s00253-006-0802-y.
Walker, M.P., Connor, E.E., Baldwin, R.L., and Kahl, S. (Year). "Ileal Tight Junction Gene Expression in Glucagon-like Peptide 2-treated Dairy Bull Calves with and without Coccidiosis", in: *2014 ADSA-ASAS-CSAS Joint Annual Meeting*: Asas).
